# Supplementary material for: Angiogenic Factors produced by Hypoxic Cells are a leading driver of Anastomoses in Sprouting Angiogenesis–a computational study
Source: Sci Rep. 2018 Jun 7;8:8726. doi: 10.1038/s41598-018-27034-8 (PMC5992150; doi:10.1038/s41598-018-27034-8)
Supplement: Supplementary file 1 — Supplementary Information [file 41598_2018_27034_MOESM1_ESM.pdf]

Supplementary Information for: "Angiogenic Factors produced by Hypoxic Cells are a leading driver of Anastomoses in Sprouting Angiogenesis – a computational study"

Maurício Moreira-Soares[1], Rita Coimbra[2], Luís Rebelo[3], João Carvalho[1], Rui D. M. Travasso[1]

1 - CFisUC, Department of Physics, University of Coimbra, Coimbra, Portugal

2 - Institute for Research in Biomedicine (iBiMED), Department of Medical Sciences, University of Aveiro, Aveiro, Portugal

3 - Department of Physics, Faculty of Sciences, University of Lisbon, Lisbon, Portugal

S1. Angiogenesis Dynamics

In the present model, the vessels are described by an order parameter $\phi(\mathbf{r}, t)$ and the cells in hypoxia by a second order parameter $\psi(\mathbf{r}, t)$, following [1, 2]. The three phases of the system (the extra cellular matrix (ECM), the vessel cells and the tissue cells) have specific values for the order parameters ϕ and ψ (see Fig. 1 of the main document). The tissue cells will not alter their shape and the modelling is focused on the evolution of the capillary.

According to the phase-field formalism, we start from a phenomenological free-energy, $F[\phi, \psi]$, given by [2–4]

$$F[\phi, \psi] = \int \left[\frac{\varepsilon^2}{2} (\nabla\phi)^2 + \frac{\varepsilon_{\mathrm{h}}^2}{2} (\nabla\psi)^2 + V(\phi, \psi) \right] \mathrm{d}^d\mathbf{r} \ . \tag{1}$$

In this free energy we include the surface tensions of the different interfaces in the system (proportional to ε and to ε_{h}), and a phenomenological potential

$$V(\phi, \psi) = -\frac{\phi^2}{2} + \frac{\phi^4}{4} - \frac{\psi^2}{2} + \frac{\psi^4}{4} + \frac{\gamma}{3}(\phi + 1)^2(\phi - 2)(\psi + 1)^2(\psi - 2) \ , \tag{2}$$

which guarantees that the minimum energy is obtained in the three phases of the system: the vessel (with $\phi = 1$ and $\psi = -1$), the ECM (with $\phi = -1$ and $\psi = -1$), and the tissue cells (with $\phi = -1$ and $\psi = 1$).

The parameters ε and ε_{h} in (1) are the widths of the interfaces and are proportional to their surface tensions. The last term of the potential $V(\phi, \psi)$ guarantees that there is an increase in energy when the vessel overlaps the tissue cells, and γ represents the energy cost for this overlap; we set this parameter high enough in order to avoid appreciable overlap. Due to the large value of γ in our simulations, the system only has interfaces between ECM and the vessels and between the tissue cells and the ECM.

The evolution of the vessel is then obtained by calculating the system's free energy functional derivative with respect to ϕ, according to [1]:

$$\frac{\partial\phi}{\partial t} = M\nabla^2 \frac{\delta F}{\delta\phi} + \text{prolif.} = M\nabla^2 \left[\phi^3 - \phi - \varepsilon^2\nabla^2\phi + \gamma(\phi^2 - 1)(\psi - 2)(\psi + 1)^2 \right] + \text{prolif.} \tag{3}$$

This equation becomes Equation (1) of the main text once the proliferation term is replaced.

The angiogenic factor in the model is modelled as a diffusive field T, that is consumed by the endothelial cells (ECs) with the rate α_T:

$$\partial_t T = D\nabla^2 T - \alpha_T \phi T \Theta(\phi) \ , \tag{4}$$

where Θ is the Heaviside step function.

A new tip cell agent is introduced at a site where there is a minimum angiogenic factor concentration T_c, a minimum angiogenic factor gradient G_m, if the site for the candidate is located at a distance larger than $4R_c$ from all other tip cell agents (due to Delta-Notch signalling) and if it is at a minimum distance of R_c from the ECM. All the activated tip cells migrate by chemotaxis, and their velocity is proportional to the angiogenic factor gradient:

$$\mathbf{v} = \chi\nabla T \left[1 + \left(\frac{G_M}{G} - 1 \right) \Theta(G - G_M) \right] \ , \tag{5}$$

where $\mathbf{v}$ is the agent velocity, χ is the chemotactic response, G_M is the gradient modulus for which the maximum velocity is attained, and $G \equiv |\nabla T|$. An tip cell is not able to enter inside a tissue cell (independently if it is in hypoxia or not): when the distance between the tip cell and a tissue cell is lower than $2R_c$, then the radial component of the velocity is set to zero and the tip cell moves in the axial direction around the tissue cell.

To link the phase-field approach described in Equation (3) to the agent-based model we set the order parameter value inside the tip cell agent to a constant value ϕ_c that depends on the tip cell velocity $|\mathbf{v}|$ and on the local ECs proliferation rate $\alpha_p(T)$ [1]:

$$\phi_c = \frac{\alpha_p(T)\pi R_c}{2|\mathbf{v}|} \ . \tag{6}$$

To improve the computational efficiency, we do not include angiogenic factor consumption inside the tip cells. As the tip cell is very small, the VEGF gradient is not significantly altered in such a small distance, and we opted to simplify the computation by measuring the gradient at a single point: the cell center. Preforming a single measurement of the VEGF gradient saves time (specially in 3D) that would be used in calculating several interpolations to obtain the VEGF concentrations at the cell boundary and averaging over all of the differences of concentrations. Since the amount of VEGF consumed by the tip cells would be very small when compared to the VEGF consumed by all the cells in the network, this choice does not change in a significant way the total amount of VEGF in the system, it is able to obtain the correct gradient, and it is faster.

The blood is able to flow through the neo-vessels, once there are anastomoses in the vascular network. In order to calculate the regions irrigated in the tissue, we introduce a simple system to identify the vessels where blood is flowing. We do not model explicitly the oxygen diffusion in the simulation because the oxygen's consumption rate is sufficiently high that it reaches the stationary state much faster than any other relevant process in the simulation [5]). We start by extracting the medial lines of the vascular structure, thus finding the bifurcations sites and the length of every vessel. The extraction method used for this purpose is detailed in [6] and consists in the application of a set of topological masks that determines if a point is a simple point (marked to be removed) or an essential point (part of the final skeleton). After some iterations the skeleton of the blood vessel network is obtained (as seen in Fig. 1).

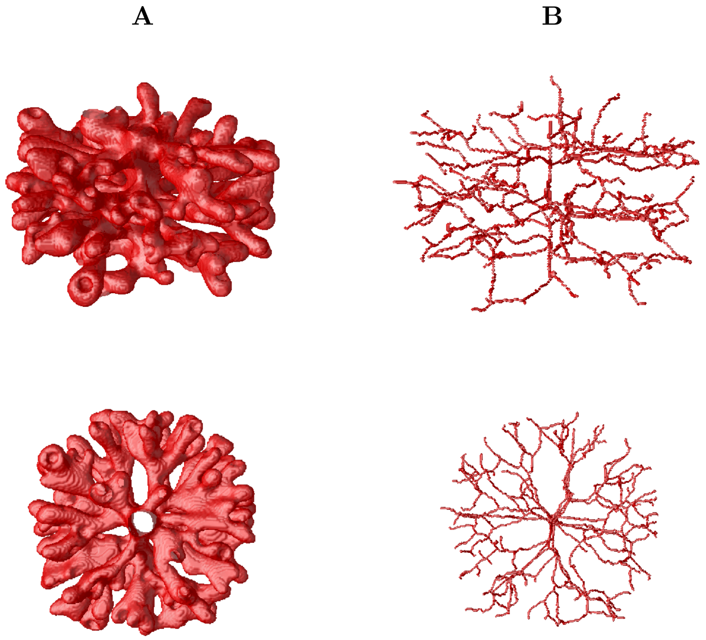

Figure 1. The column **A** shows the vessel network before the thinning algorithm from two different viewing angles and the column **B** shows the skeleton after the application of the algorithm.

Afterwards we calculate the blood flow rate in every vessel using the Hagen-Poiseuille law:

$$Q = K \frac{R^4}{L} \Delta p \ , \tag{7}$$

where Δp is the difference in pressure between the beginning and end of every vessel, L the vessel length, R the vessel radius, Q is the flow rate, and K is a constant proportional to the inverse of the blood viscosity. The value of K was set to $K = 1$, since we are only interested in identifying the vessels with $Q \neq 0$, and not the precise value of the flow rate. In the simulation there is a single input node (at the top of the simulation box) and a single exit node (at the bottom of the simulation box).

S2. Model Parameters

The parameters for the model were obtained, when possible, from experimental data available on the literature and are presented in Table 1.

Table 1. Values of the parameters used in the simulations.

Parameter	Description	Value *in Silico*	*in Vivo*
a	Lattice Parameter	1	1.25 μm
Δt	Unity of time	1	25 min
R_c	Cell Radius	4	5 μm [7,8]
D	Diffusion coefficient of VEGF	100	10^{-13} m^2/s [9,10]
M	Endothelial Cell Mobility	1	10^{-15} m^2/s [9,10]
d	Oxygen Diffusion Length	20	25 μm [11]
β_p	Proliferation rate	$0.6 \sim 2.5$	—
$\alpha_T = D/R_c^2$	Consumption rate of VEGF	6.25	0.004 s^{-1} [1]
ε, ε_h	Interface width	1	1.25 μm
γ	Energy cost for overlap	10	—
χ	Chemotactic Response of ECs	$100 \sim 2500$	—
T_s	[VEGF] at the source	1	—
T_p	[VEGF] for maximum proliferation	0.3	—
T_c	[VEGF] for branching	0.055	—
G_M	$\lvert\nabla$VEGF$\rvert$ for max. velocity	0.03	0.0375 a.u/μm
G_m	$\lvert\nabla$VEGF$\rvert$ for branching	0.01	0.0125 a.u/μm

S3. Computational Methods

The simulations of the angiogenesis model were performed on parallel task farm on the computer cluster of the Centre of Physics of the University of Coimbra (see example of simulation in Figure 2). The gradients and laplacians were calculated using the finite differences over a discrete mesh with dimensions: $200 \times 200 \times 100$ for the 3D simulation and 300×300 for the 2D, with the lattice parameter $a = 1$. In the 3D simulations were employed periodic boundary conditions on the radial direction and Neumann boundary conditions on the axial direction (no flux condition). The 2D simulations were performed with no flux conditions along both x and y directions. The initial conditions contain in one main vessel placed at the center (border) of the 3D (2D) simulation box and randomly distributed hypoxic cells at a distance larger than 25 μm of the main vessel (about 80 cells in the 2D simulations and 240 cells in the 3D simulations). The order parameter $\psi(\mathbf{r}, t)$ was integrated in time before the start of the simulation in order to determine the diffuse interface between the hypoxic cells and the ECM. The process used to obtain the pressure at every bifurcation (needed to calculate the blood flow rate from equation (7)), leads to a large sparse matrix which requires the application of an efficient method to invert matrices. The method of choice was the PLU decomposition with partial pivoting, implemented in the ScaLAPACK [12].

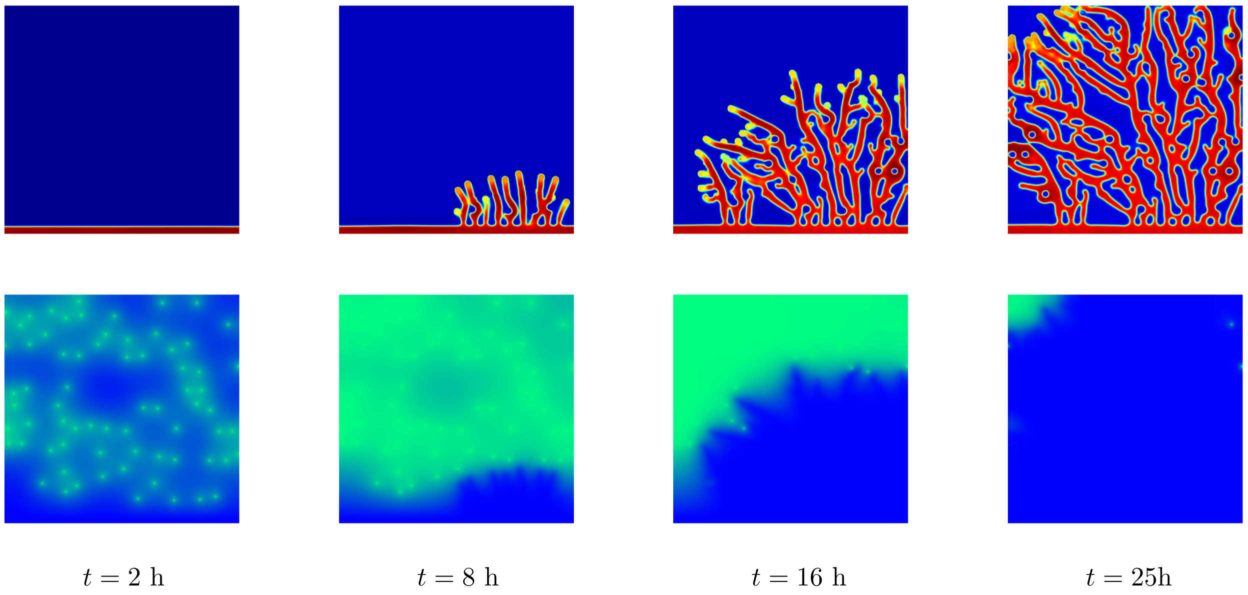

Figure 2. Example of vessel growth with Rule 1: In this figure we show the evolution of the vascular morphology (top row) and of the corresponding VEGF concentration (bottom row) produced by randomly distributed hypoxic cells for a network growing under Rule 1. The value of the VEGF concentration at the hypoxic cells is maintained at T_s (in light green) and reaches zero (in blue) inside the irrigated regions.

S4. Variable chemotaxis response

The chemotactic response of the tip cells depends on the amount of VEGF receptor 2 (VEGFR2) molecules at their cytoplasm membrane. However, it is known that the expression of VEGFR2 is lower in hypoxic environments [13, 14], leading to a lower chemotactic response. Here we address if this fact alters the conclusions drawn regarding how the numbers of branches and anastomoses depend on the maximum tip cell velocity. With this aim, we run the 2D system for both rules, but with the following altered tip cell velocity:

$$\mathbf{v} = \chi_T(T)\nabla T \left[1 + \left(\frac{G_M}{G} - 1\right)\Theta(G - G_M)\right] , \tag{8}$$

where

$$\chi_T(T) = \chi \left[1 + \left(\frac{1-T}{1-T_p} - 1\right)\Theta\left(T - T_p\right)\right] . \tag{9}$$

In our model, when the tissue cells are not irrigated they will produce VEGF, and therefore we will use the concentration of VEGF as a proxy to quantify hypoxia. In this way, the new term $\chi_T(T)$ that is included in this equation for the tip cell velocity implies that the chemotactic response decreases with hypoxia (See Figure 3).

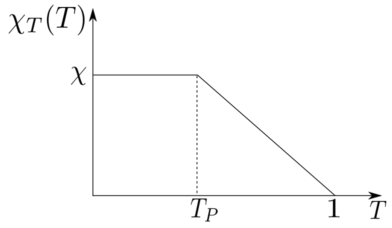

Figure 3. Variable Chemotactic Response: The chemotactic response decreases linearly with the VEGF concentration T for values higher than the VEGF concentration for maximum proliferation T_p.

For simulations with Rules 1 and 2, we have varied the value of χ and quantified the density of anastomoses and branches as well as the branches' diameter (see Figure 6).

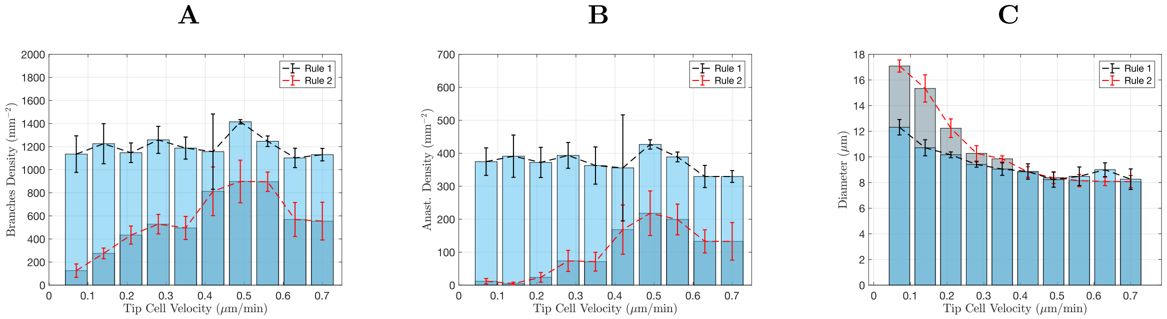

Figure 4. Characterization of the vascular networks in with variable chemotactic response. Comparison of the branch density (A), anastomoses density (B) and average vessels' diameter (C), between the two rules implemented in the model, as a function of the maximum tip cell velocity. The networks were analyzed after they had grown sufficiently to deactivate the VEGF production in all tissue cells. For each point we plot the average of three simulations.

We observed even more striking results than in Figure 4 of the main text. Namely, Rule 1 leads to a clear increase in the number of branches and anastomoses, but at the same time, to much more stable vascular networks (regarding branches and anastomoses densities as well as vessel diameters). For Rule 2, as the tip cell velocity increases, the number of branches increases until a maximum is reached, and the density of anastomoses follows, qualitatively, the same non-monotonic behaviour. For Rule 1 the number of branches and anastomoses are significantly higher and their relative variations with tip cell velocity are very small. As in Figure 4 of the main text, the vessel diameter decreases with the tip cell velocity, but now it is clear that Rule 1 leads to a smaller variation of the vessel diameter. We conclude that the results described in the main text are robust to the inclusion of variable chemotactic response.

S5. Growth factor degradation

In the model in the main text only the VEGF uptake by the vessel cells was considered and not the VEGF degradation. However, the conclusions drawn are not altered when we add VEGF degradation. The degradation rate that is observed in vivo is of the order of $\lambda_{\text{deg}} = 1.0 \times 10^{-4}\ \text{s}^{-1}$ [15]. This degradation rate, together with the VEGF diffusion constant, leads to a typical VEGF diffusion length of 30 μm, slightly larger than the oxygen diffusion length.

With VEGF degradation, equation (2) of the main document is replaced by

$$\frac{\partial T}{\partial t} = D\nabla^2 T - \alpha_T \phi T \Theta(\phi) - \lambda_{\text{deg}} T \Theta(-\phi) \ . \qquad (10)$$

We simulated the vascular growth as a function of the endothelial cell proliferation rate for Rule 1 and for Rule 2. The corresponding branches and anastomoses densities are plotted in Figure 5.

These results were obtained with the same parameters as in the main text, except for the value of T_s which was doubled (when introducing degradation in the model the total supply of VEGF decreased, and we increased T_s so that the VEGF concentration at the vessels was still large enough to induce sprouting).

As for the case without VEGF degradation, we observe that Rule 1 leads to vascular morphologies much more independent of EC proliferation and with higher number of anastomoses.

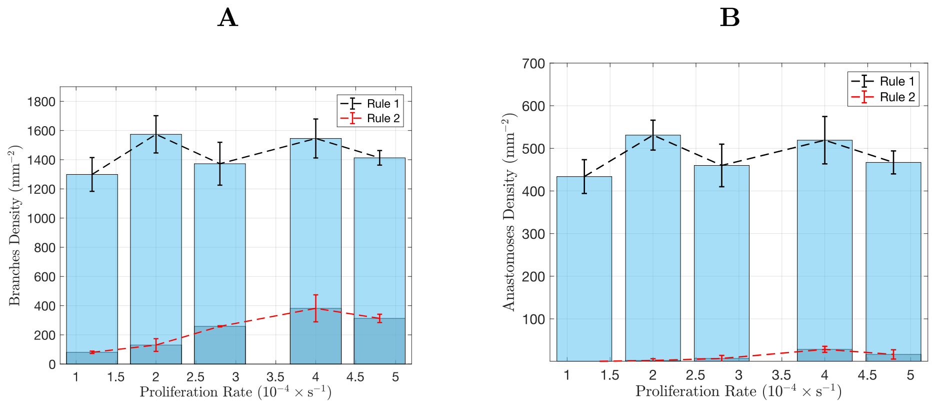

Figure 5. Characterization of the vascular networks in the presence of VEGF degradation: Comparison of the branches density (A) and the anastomoses density (B) for the two rules implemented in the model, as a function of the maximum proliferation rate. The networks were analyzed after they had grown enough to deactivate the VEGF production in all tissue cells. The error bars represent the uncertainty in the average of 3 simulations (for each condition).

S6. Variable hypoxic cell density

In order to address the question of how the number of vessels depends on the number of cells in hypoxia, we have performed simulations where the chemotactic velocity and the proliferation rate were set as constants, and the density of hypoxic cells was varied. Below we show, for 2D, that the number of branches and the number of anastomoses increase linearly with the number of cells in hypoxia for Rule 1. Again, we observe a stronger trend with Rule 1 for the number of vessels to follow the tissue demand: more cells in hypoxia lead to a higher vessel density.

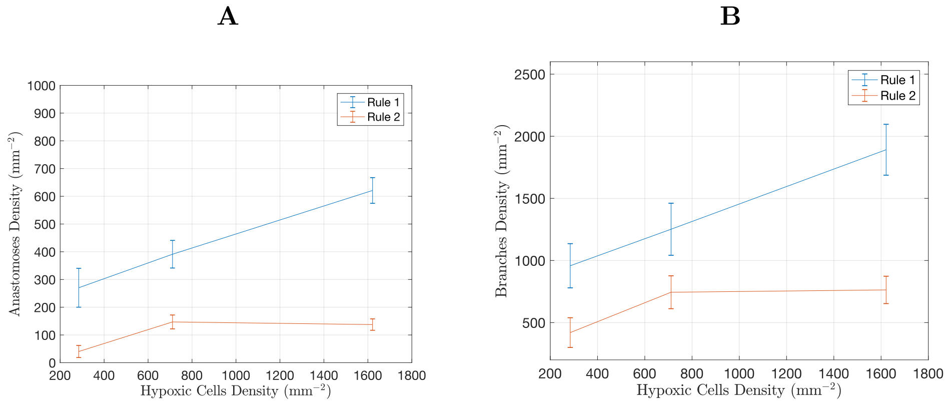

Figure 6. Characterization of the vascular networks for different number of cells in hypoxia. Densities of branches (A) and anastomoses (B) as a function of the hypoxic cells density in the tissue. For Rule 1 the two quantities show a linear relation with the number of cells in hypoxia, while for Rule 2 we observe a saturation of the densities of branches and anastomoses. These densities are much higher for Rule 1 than for Rule 2, in accordance with the results presented in the manuscript.

S7. Varying the consumption rate α_T

We run 2D simulations to predict how the morphologies of 2D vascular networks depend on EC proliferation rate for different values of VEGF consumption rate α_T. In particular, we measured the anastomoses density for α_T values one third lower and 50% higher than the value used in the main simulations in the manuscript. The results are presented in Figure 7.

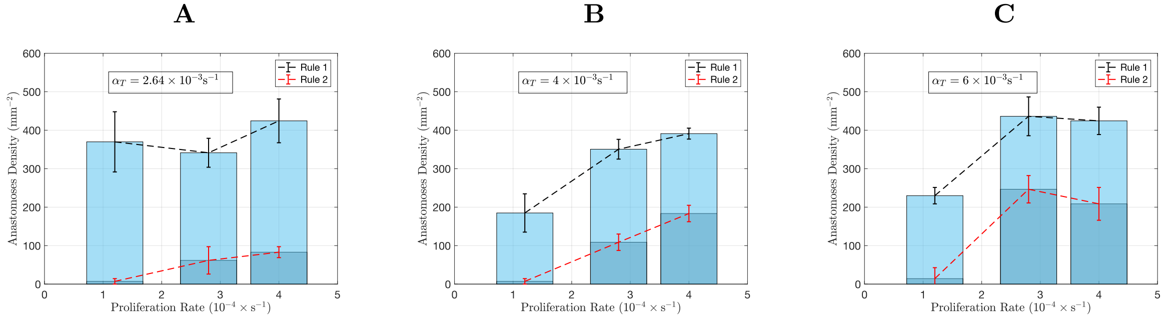

Figure 7. Anastomoses density as a function of the proliferation rate α_p for three values of α_T: The values of α_T in panels A, B and C are respectively $\alpha_T = 2\alpha_{T,0}/3 = 2.64 \times 10^{-3}$ s^{-1}, $\alpha_T = \alpha_{T,0} = 4.0 \times 10^{-3}$ s^{-1} and $\alpha_T = 3\alpha_{T,0}/2 = 6.0 \times 10^{-3}$ s^{-1}, where $\alpha_{T,0}$ is the consumption rate used in the main document. We observe that for the three consumption rates, Rule 1 always leads to more anastomoses and to more resilient networks than with Rule 2.

For the three VEGF consumption rates, we observe that Rule 1 leads to vascular morphologies with a higher number of anastomoses than Rule 2. Moreover, similarly to what is observed in the main document, the anastomoses density in the networks that grow under Rule 1 has a much smaller relative variation with EC proliferation rate than the anastomoses density in the networks that grow under Rule 2. Therefore, the results observed and the conclusions drawn in the main document are robust to variations in the VEGF consumption rate.

References

1. Travasso, R. D. M., Corvera Poiré, E., Castro, M., Rodríguez-Manzaneque, J. C. & Hernández-Machado, A. Tumor angiogenesis and vascular patterning: A mathematical model. *PLoS ONE* **6**, e19989 (2011).

2. Nonomura, M. Study on multicellular systems using a phase field model. *PLoS ONE* **7**, e33501 (2012).

3. Emmerich, H. Advances of and by phase-field modelling in condensed-matter physics. *Adv. Phys.* **57**, 1–87 (2008).

4. Travasso, R. D. M., Castro, M. & Oliveira, J. C. R. E. The phase-field model in tumor growth. *Philos. Mag.* **91**, 183–206 (2011).

5. Secomb, T. W., Alberding, J. P., Hsu, R., Dewhirst, M. W. & Pries, A. R. Angiogenesis: an adaptive dynamic biological patterning problem. *PLoS Comput. Biol* **9**, e1002983 (2013).

6. Palágyi, K. & Kuba, A. A 3D 6-subiteration thinning algorithm for extracting medial lines. *Pattern Recognit. Lett.* **19**, 613–627 (1998).

7. Carlson, K., Bourne, W., McLaren, J. & Brubaker, R. Variations in human corneal endothelial cell morphology and permeability to fluorescein with age. *Exp. eye research* **47**, 27–41 (1988).

8. Gebb, S. & Stevens, T. On lung endothelial cell heterogeneity. *Microvasc. research* **68**, 1–12 (2004).

9. Ristimäki, A., Narko, K., Enholm, B., Joukov, V. & Alitalo, K. Proinflammatory cytokines regulate expression of the lymphatic endothelial mitogen vascular endothelial growth factor-c. *J. Biol. Chem.* **273**, 8413–8418 (1998).

10. Schugart, R. C., Friedman, A., Zhao, R. & Sen, C. K. Wound angiogenesis as a function of tissue oxygen tension: a mathematical model. *Proc. Natl. Acad. Sci.* **105**, 2628–2633 (2008).

11. Grote, J. Tissue respiration. In *Human physiology*, 508–520 (Springer, 1983).

12. Blackford, L. S. *et al. ScaLAPACK Users' Guide* (Society for Industrial and Applied Mathematics, Philadelphia, PA, 1997).

13. Ulyatt, C., Walker, J. & Ponnambalam, S. Hypoxia differentially regulates vegfr1 and vegfr2 levels and alters intracellular signaling and cell migration in endothelial cells. *Biochem. biophysical research communications* **404**, 774–779 (2011).

14. Nevo, O., Lee, D. K. & Caniggia, I. Attenuation of vegfr-2 expression by sflt-1 and low oxygen in human placenta. *PloS one* **8**, e81176 (2013).

15. Serini, G. *et al.* Modeling the early stages of vascular network assembly. *The EMBO journal* **22**, 1771–1779 (2003).
